# Assessment of the integrality of the psychosocial care network: multiple case study, Southern Region of Brazil, 2021

**DOI:** 10.1590/S2237-96222026v35e20240890.en

**Published:** 2026-07-27

**Authors:** Larissa Weber, Claudia Flemming Colussi

**Affiliations:** 1 Prefeitura Municipal de Florianópolis Florianópolis Florianópolis SC Brasil Prefeitura Municipal de Florianópolis, Florianópolis, SC, Brasil; 2 Universidade Federal de Santa Catarina Florianópolis Florianópolis SC Brasil Universidade Federal de Santa Catarina, Florianópolis, SC, Brasil

**Keywords:** Integrality in Health, Health Evaluation, Mental Health, Community Networks, Case Reports, Integralidad en Salud, Evaluación en Salud, Salud Mental, Redes Comunitarias, Informes de Casos

## Abstract

**Objective:**

To assess the integrality of health actions of the services in the Psychosocial Care Network (Raps) in three municipalities in the Southern Region of Brazil, with different population sizes and varied Raps configurations.

**Method:**

Evaluative research based on a multiple case study. An analysis and judgment matrix was used, comprising two dimensions, six subdimensions, and 16 indicators. Primary data from five interviews per municipality with managers, professionals, and users, totaling 15 interviews, were cross-analyzed with data from direct observation and secondary data, all collected in the field in 2021. Content analysis was applied to the qualitative data, whereas quantitative data were submitted to descriptive analysis.

**Results:**

The three cases showed satisfactory results in the “Essential elements” dimension. This finding highlighted the feasibility of implementing qualified actions for territorial care, such as providing receptiveness, emphasizing users’ uniqueness and autonomy, and supporting family members, regardless of the size of municipalities’ Raps. Only case 3 performed well in the “Integrations” dimension, while the other two achieved fair performance. In a scenario where all Raps components coexist, the presence of work processes aligned with the logic of network-based care favored the performance of case 3 in this dimension.

**Conclusion:**

The provision of singular and territorial care stood out positively in the three cases. It was also shown necessary to improve the articulation of Raps components, especially through investments in the production, integration, and dissemination of data.

## Resumen

**Objetivo:**

Evaluar la integralidad de las acciones en salud de los servicios de la red de atención psicosocial (Raps) en tres municipios de la región Sur de Brasil con diferentes tamaños poblacionales y variadas configuraciones de la Raps.

**Método:**

Investigación evaluativa basada en un estudio de casos múltiples. Se utilizó una matriz de análisis y juicio con dos dimensiones, seis subdimensiones y 16 indicadores. Los datos primarios provinieron de cinco entrevistas por municipio con gestores, profesionales y usuarios, totalizando 15 entrevistas, y fueron triangulados con datos obtenidos mediante observación directa y datos secundarios, todos recolectados en campo en 2021. Se realizó análisis de contenido de los datos cualitativos, mientras que los datos de naturaleza cuantitativa fueron sometidos a análisis descriptivo.

**Resultados:**

Los tres casos obtuvieron resultados satisfactorios en la dimensión “Elementos esenciales”. Este hallazgo evidenció la viabilidad de implementar acciones calificadas de cuidado territorial, como la oferta de acogida, el énfasis en la singularidad y autonomía de los usuarios, y la atención dirigida a los familiares, independientemente del tamaño de la Raps de los municipios. Solo el Caso 3 fue bien evaluado en la dimensión “Integraciones”, en la que los otros dos casos tuvieron desempeño regular. La presencia de procesos de trabajo alineados con la lógica del cuidado en red en un escenario donde coexisten todos los componentes de la Raps favoreció el desempeño del Caso 3 en esta dimensión.

**Conclusión:**

Sobresalió positivamente la oferta de cuidado singular y territorial en los tres casos. Asimismo, se evidenció la necesidad de perfeccionar la articulación de los componentes de la Raps, especialmente mediante inversiones en la producción, integración y difusión de datos.

**Ethical aspects**

This research respected ethical principles, having obtained the following approval data:

Research ethics committee Opinion number, Approval date, Certificate of submission for ethical appraisal

Universidade Federal de Santa Catarina 4.624.644, 31/3/2021, 43341921.5.0000.0121

Secretaria Municipal de Saúde de Curitiba 4.791.120, 18/6/2021, 43341921.5.3001.0101

Informed consent form Obtained from all participants prior to data collection.

## Introduction

Integrality presupposes the user as the central element of health care and requires the adoption of collaborative and coordinated practices to promote, prevent, restore health, and rehabilitate individuals (1), recognizing the interdependence among mental health, physical health, and social well-being (2). In the field of psychosocial care, integral care involves developing singular therapeutic projects that understand individuals within their family, social, political, and historical contexts, as well as the composition of an integrated, qualified care network (3). 

The Psychosocial Care Network (*Rede de Atenção Psicossocial*, Raps), which consists of a thematic health care network (4), was established in Brazil based on this principle. It seeks to expand user access, diversify care strategies, and articulate points of care. These are grouped into the following components: primary health care, specialized psychosocial care, emergency and urgent care, hospital care, residential care of a transitional nature, deinstitutionalization, and psychosocial rehabilitation (5).

This scenario of reorientation of the care model still lacks evaluative research, especially focused on the integration among Raps services (6,7). The proposal for evaluations within health networks is still incipient and challenging, due to the complexity posed by analyzing interactions among multiple actors and services (8). 

This study considered the potential of evaluation to support decision-making and improve actions, services, networks, and health systems. It is also considered that the organization of Raps will not occur in a standardized manner across the Brazilian territory, given municipal and regional particularities. The objective of this study was to assess the integrality of health actions provided by Raps services in three municipalities in the Southern Region of Brazil with different population sizes and Raps configurations.

## Methods Design

This is a cross-sectional study, characterized as evaluative research based on a multiple-case study (9) involving three municipalities. The evaluation was conducted through the application of an analysis and judgment matrix developed to assess the actions and services of psychosocial care from the perspective of integrality in municipalities, in order to provide means to qualify care in the field, management, and social participation. This study did not intend to evaluate the instrument (the matrix) itself, but rather the three selected cases. 

The matrix consisted of two dimensions (D) and six subdimensions (S): “D1 Essential elements,” subdivided into “S1 Structure,” “S2 Care,” and “S3 Management”; and “D2 Integrations,” subdivided into “S4 Care integration,” “S5 Functional integration,” and “S6 Normative integration,” totaling 16 indicators and 52 measures (10). The original proposal underwent some changes: merging of the measures “M4 Primary health care services” accessible to people with disabilities and “M5 Psychosocial Care Center (*Centros de Atenção Psicossocial*, Caps)” accessible to people with disabilities, because municipalities would be penalized in the assessment if any of the services were not accessible; merging of “M18 Development of the Individualized Therapeutic Plan (*Plano Terapêutico Singular*, PTS) in primary health care for cases followed in matrix support” and “M19 Scheduling of home visits by primary health care teams for cases discussed in matrix support,” motivated by the understanding that the scheduling of visits is part of singular therapeutic projects, with no need to separate this action into two measures; and exclusion of “M17 Proportion of users with medication-centered treatment,” given that it would not be feasible to extract this data from service records.

### Setting

The selection of cases was based on population size and the composition of municipalities’ Raps (Table 1). At least two of the following criteria were also considered.

**Table 1:** Prerequisites for the selection of cases in the Psychosocial Care Network (Raps). Southern Region of Brazil, 2021 (n=3)

Prerequisites	Case 1	Case 2	Case 3
Population size (inhabitants)	Up to 20 thousand	70–150 thousand	>150 thousand
Raps service composition	Provide mental health care offered predominantly by primary health care. Do not have a Psychosocial Care Center (Caps).	Have at least one Caps and another service among the following Raps components: residential care of a transitional nature, rehabilitation, or deinstitutionalization.	Include all Raps service components.

To obtain a satisfactory result in the survey “Assessment of mental health actions in primary health care,” which used the database of Brazil’s National Program for Improving Primary Health Care Access and Quality (*Programa de Melhoria do Acesso e da Qualidade da Atenção Básica*, PMAQ-AB) (11). The municipalities were divided by population size, and the value corresponding to the upper quartile was calculated for each of the three groups to identify the best performers. Mandatory criterion for selecting case 1.
To participate in the project “Training pathways in Raps: exchange between experiences,” promoted by the Ministry of Health, whose objective was to foster the development of Raps by conducting exchange activities among professionals from different municipalities and hiring network facilitators.
To present low hospitalization rates resulting from mental health conditions between September 2019 and August 2020. To calculate the rate, the estimated population of the municipalities in the Southern Region in 2019 and the total number of hospitalizations due to mental and behavioral disorders resulting from the use of alcohol or other psychoactive substances; schizophrenia, schizotypal and delusional disorders; mood disorders; neurotic and stress-related disorders; intellectual disability; and other mental and behavioral disorders were extracted from the Information Brazilian Unified Health System Information Technology Department (*Departamento de Informática do Sistema Único de Saúde*, DATASUS). The municipalities were separated by population size to identify those in the lower quartile in each group, making them eligible.

After applying these prerequisites and criteria, 24 municipalities in the Southern Region were eligible for case 1; four for case 2; and four for case 3. The final selection considered representation across the three states, leading to the decision to choose one case per state.

### Participants

One manager, two professionals, and two Raps users from each of the three municipalities participated in the study, for a total of 15 participants. Managers and professionals were selected by the management of municipal health departments, except for one professional, who was selected by convenience. The interviewed managers and professionals indicated users.

### Variables

Outcome variables and explanatory variables were used. Outcome variables included the completeness of Raps actions and services in the municipalities studied, as measured by the matrix scores. The explanatory variables considered the municipality’s population size (small, medium, large) and the composition of the local Raps (presence or absence of Caps and other points of care).

### Data sources and measurement

Primary data collection included interviews and observations conducted in July and August 2021. In case 3, one region of the city was designated for data collection. All sites visited provided direct services, were accredited, and had full teams at the time of data collection.

Semistructured interviews were conducted mostly in person (n=14). They were carried out by a single researcher using a script based on the matrix’s elements. All interviews were recorded and transcribed, with participants’ consent, and identified by category (G=manager, P=professional, U=user), followed by a number from 1 to 6.

Systematic observation was conducted by the same researcher who carried out the interviews and took place at the same sites, totaling three shifts per municipality. Among other aspects, structure, interactions, and information displayed at service reception areas were observed. Data were recorded in a field diary and used as complementary input in the analysis of some measures.

Secondary data were collected in the field from documents from 2020–2021 related to the Raps components in the municipalities, including protocols, flowcharts, regulations, meeting minutes, memoranda, and planning instruments. Documents were represented by the letter D, followed by a number from 1 to 5.

### Bias control

Participant indication may have favored professionals and users who were more engaged or had positive experiences, potentially introducing selection bias. In the case of users, gratitude bias may have inhibited them from making criticisms of the services. 

The use of recorded interviews and in-person observations may elicit socially desirable responses that obscure service weaknesses, particularly among professionals and managers, thereby leading to information bias. 

Conducting interviews and observations by a single researcher may lead to interpretation bias or other conduct bias, even when using standardized scripts. Efforts were made to minimize this potential bias through data convergence and validation.

### Study size

The study involved three municipalities in the Southern Region of Brazil and included 15 participants, five per municipality.

### Statistical methods

Qualitative data were subjected to content analysis (12), using the predefined subdimensions and dimensions of the matrix as categories. Quantitative data were transferred to Excel spreadsheets for descriptive analysis. Given the diversity of sources and data collection methods, methodological triangulation was chosen as the analytical model (13), resulting in both separate analyses of each case and an integrated analysis in which similar and divergent aspects were examined.

In the matrix, the sum of the measure scores resulted in the score for each indicator. The obtained score (OS) was divided by the maximum possible score of the indicator (MS), using the formula: “score=(∑OS/∑MS)×100.” The resulting percentage was interpreted as follows: 0–49% was poor; 50–74% was fair; and 75% or above was good. For the analysis of subdimensions, the following scores were assigned to indicators: 1 (one) for good; 0.5 (half) for fair; and 0 (zero) for poor. The indicator values in the subdimensions were summed to obtain their total score. The same formula and interpretation applied to indicators were used for subdimensions. This procedure was also applied to the dimensions—by summing each subdimension’s score and applying the formula, the final judgment was assigned.

## Results

Table 2 characterizes the participating professionals and managers. As for the users, they were four men and two women who had been receiving treatment for at least a year and a half. The cases were characterized in terms of demographic data (14) and Raps composition (Table 3).

**Table 2:** Characteristics of health professionals and managers interviewed in the three municipalities. Southern Region of Brazil, 2021

Code	Categories	Sex	Training	Place of practice	Length of service in the municipality
G1	Manager	Female	Occupational Therapist	Psychosocial Care Center (Caps)	11 years
G2	Manager	Female	Psychologist	Mental Health Coordination	19 years
G3	Manager	Female	Nurse	Municipal Health Department	20 years
P1	Health Professional	Female	Occupational Therapist	Caps II and Caps ad	1 year and 7 months
P2	Health Professional	Female	Psychologist	Expanded Family Health Center (Nasf)	19 years
P3	Health Professional	Female	Nursing Technician	Caps II	8 years
P4	Health Professional	Female	Nurse	Health Center (UBS)	3 years
P5	Health Professional	Male	Psychologist	Nasf	2 years
P6	Health Professional	Female	Psychologist and Acupuncturist	Family Health Strategy (ESF)	7 years

Based on data from interviews, observations, and documents, all three cases achieved satisfactory performance in the “D1 Essential elements” dimension. The “D2 Integrations” dimension was considered satisfactory in case 3 and fair in the others. 

The scores obtained by each municipality after application of the matrix are presented in Table 4. Case 1 achieved maximum performance in “S1 Structure,” “S2 Care,” and “S6 Normative integration,” and had high scores in measures related to primary health care (14 out of 15).

**Table 4:** Evaluation results of the three cases based on the scores of indicators (I), subdimensions (S), and dimensions (D) of the analysis and judgment matrix. Southern Region of Brazil, 2021

Indicators	Case scores			Subdimensions	Case scores			Dimensions	Case scores		
	1	2	3		1	2	3		1	2	3
I1 Physical resources	100	58	75	S1 Structure	100	75	100	D1 Essential elements	100	100	100
I2 Human Resources	100	100	100								
I3 Receptiveness	100	100	100	S2 Care	100	100	100				
I4 Diverse follow-up	100	100	100								
I5 Crisis care	100	100	100								
I6 Continuing Health Education (Educação Permanente em Saúde, EPS)	67	60	80	S3 Management	83	83	100				
I7 Management tools	100	100	100								
I8 Social control	100	75	100								
I9 Shared responsibility for cases	80	100	67	S4 Care integration	83	100	83	D2 Integrations	67	67	100
I10 Promotion and prevention in the territory	83	83	83								
I11 Rehabilitation in the territory	67	83	100								
I12 Health surveillance	67	62	62	S5 Functional integration	33	50	83				
I13 Clinical management	50	75	75								
I14 Network articulation	37	37	75								
I15 Regulation	100	33	100	S6 Regulatory integration	100	50	100				
I16 Computerization	75	50	75								

Investments in community health agents—”we can achieve this integrality through the information brought by the health agent” (G3)—in the implementation of multidisciplinary teams—”The Expanded Family Health Center (*Núcleo Ampliado de Saúde da Família*, Nasf) makes a wonderful difference, (...) with this proposal to make connections, (...) flow design, referral, matrix support” (G3)—and in integrative and complementary practices—”offering (...) auriculotherapy, reiki” (G3)—demonstrated good performance in this set of measures.

On the other hand, difficulties were noted in formalizing forums and meetings in the municipality (intra- and intersectoral), as reflected in the low performance in “I14 Network articulation,” which negatively impacted S5 Functional integration. Team meetings in primary health care were also not occurring during data collection.

There was a perception that case discussions could be more preventive—”usually it is done when things get complicated (...) sometimes we arrive late” (G3). The proximity among professionals sometimes minimized the designation of formal spaces for exchanges—”there could be some meeting, (...) greater integration. Of course, sometimes personal contact facilitates things” (P5). Occasional misalignment among professionals could compromise care—”sometimes it leaves a bit to be desired (...), because one says one thing, (...) the other does not agree” (U5).

The need for greater investment in child and adolescent care was raised—”there’s no soccer school, no other activity that can involve these children (...) it’s not possible to have people with a healthy mind if they are not treated healthily from childhood” (U6).

Case 2 stood out for its performance in “I9 Shared responsibility for cases,” in “S2 Care,” and in “S4 Care integration.” Despite the absence of Nasf (terminology retained in this study, despite service discontinuity, considering the data collection period), Caps’ matrix covered primary health care services in the municipality, and some teams included psychologists and psychology interns. Matrix support was valued by teams both in the care dimension—”they ask to speak with the technician who always participates in the matrix support, because they know it is appropriate” (P3)—and in the technical-pedagogical role—”it worked very well, because cases were discussed, doubts were clarified, sometimes a medication was inserted into the case discussion or an interconsultation was made” (P4).

The network’s performance was impaired by low primary health care coverage (43.0%). Considering only the Family Health Strategy (*Estratégia Saúde da Família*, ESF), coverage was even lower (21.0%). The likely overload resulting from this situation affected work processes, undermining health promotion and prevention actions. The municipality planned to increase primary health care coverage to 60.0% and ESF coverage to 40.0% by 2025, in addition to implementing health education groups in the units (D2). The approach to mental health in primary health care also required further incentives—”we managed to advance a lot in establishing Caps as public policy in the municipality, but primary health care still has a long way to go in mental health discussions” (G2).

Caps teams had a history of initiatives aimed at network articulation—”building Raps has never been very easy; this network articulation often seems like a one-way movement (...) centered on the Caps” (G2). However, the absence of an institutional schedule of network meetings at the time of data collection, even if justified by the pandemic, indicated a weak definition of work flows and actors’ roles. This was evident from the low scores in “I15 Regulation” and “I14 Network articulation”—”There is nothing formal, written, that says do this, do that” (P3). The importance of keeping flows flexible was highlighted so that the singularity of cases would not be overlooked—”The case does not exist on paper; the case exists when discussed within the team” (P3).

Case 3 was assessed as satisfactory in all subdimensions and dimensions, with maximum scores in the three subdimensions of “D1 Essential elements” and in “S6 Normative integration.” Good performance was driven by territorial and longitudinal work: “territoriality greatly underpins the work, (...) it amplifies our care capacity, (...) the bond between teams and the territory” (P2). Psychology and psychiatry professionals were designated as supporters of primary health care in the Municipal Health Plan (*Plano Municipal de Saúde*, PMS), developing intra- and intersectoral actions in a defined territory (D3). Emphasis on users’ singularity and autonomy was also noteworthy—”We already stopped one medication, reduced another, (...) at the next appointment I will say I don’t want sleeping medication anymore. (...) I’ll stay with the mood stabilizer, which I still think is necessary” (U1).

The municipality had a guiding document for mental health care, which included the responsibilities of each Raps service, referral criteria, and clinical guidelines (D4). There were also specific institutional guidelines for the Caps, aligned with the psychosocial care model (D5). 

The coexistence of formal and informal mechanisms of intra- and intersectoral articulation positively impacted integrality: “a very practical way of seeing this integrality of care is precisely this support from the network” (P1). The less satisfactory performance in the indicators for “S4 Care integration” and “S5 Functional integration” suggested that the municipality should strengthen shared responsibility and health surveillance.

The integrated analysis favored comparison among cases and highlighted convergent and divergent aspects. The good performance of the cases in most subdimensions justified the predominance of favorable aspects over unfavorable ones (Table 5).

## Discussion

The use of the matrix as an evaluative instrument revealed both strengths and weaknesses in the comprehensiveness of care within the Raps of the three municipalities in the Southern Region of Brazil. At the same time, cross-validation demonstrated convergence across the different data sources. It was possible to identify that Cases 1 and 2 achieved satisfactory results in “D1 Essential Elements” and fair performance in “D2 Integrations,” whereas Case 3 performed well in both dimensions.

Limitations of this study included the inability to visit all municipal services; the representation of professionals and managers was restricted to three professional groups; and data collection during the COVID-19 pandemic, which also led to the cancellation of the pilot study.

The good performance of the three cases in D1 corroborates the feasibility of providing qualified care in the territories, regardless of the size of municipalities’ Raps. Receptiveness, the emphasis on the singularity of users, and attention to family members are globally recommended practices (15–16) and were identified in the different evaluated scenarios. The National Mental Health Policy influenced their dissemination (*Política Nacional de Saúde Mental*, PNSM), whose regulations guide managers and workers in this direction (5,17–18).

The strength of care coordination and network organization by primary health care teams was evident in Cases 1 and 3, in line with the National Primary Health Care Policy (*Política Nacional de Atenção Básica*, PNAB) (19). In Case 2, certain responsibilities were shifted from primary health care to the Caps. This reflects the reality of other municipalities (7,2–021) and is supported by Ordinance No. 336/2002, which assigned Caps the responsibility for organizing demand and the mental health care network in the territory (18). Subsequently, the Raps Ordinance established that the coordination of care may be attributed to Caps or to primary health care (5). 

Another favorable convergence toward integrality identified in Cases 1 and 3 was the establishment of well-defined referral criteria within the network, along with the implementation of mental health regulation. Protocols and flows qualify reception and referrals, guide conduct, improve articulation between services, and prevent unnecessary hospitalizations (22), constituting a fundamental strategy for network integration (23). Given the structural differences between the two cases, this work process does not require a robust Raps implementation. The adoption of these instruments increased after the pandemic, including guidance on access to mental health care (24).

Care diversification was more pronounced in Cases 2 and 3, driven by actions aimed at psychosocial rehabilitation and deinstitutionalization through social spaces and income-generation initiatives. These services foster autonomy, citizenship, and social inclusion—interventions that are as important as clinical care for individuals experiencing psychological distress (7).

On the one hand, municipalities with leaner networks may have fewer professionals and services available to implement such actions; on the other hand, the absence of specialized mental health resources reinforces the need for active participation by managers in regional health agreements, as observed in Case 1. Regionalization increases access and system efficiency by integrating service provision through continuous, cooperative action among municipalities (25). Through this mechanism, an agreement was established between Case 1 and a neighboring municipality, enabling the operation of a regional Caps.

The presence of work processes aligned with the logic of networked care in a setting where all Raps components coexist contributed to the strong performance of Case 3, the most successful in this evaluation. Among these processes, the systematization of meetings and articulation forums enhanced collaboration among professionals, resulting in the best assessment of “D2 Integrations.” Collaborative work is especially productive in the Raps context, as it breaks with a fragmented care model by valuing professional competencies, horizontal communication, and shared decision-making (26).

The absence of users in these psychosocial-care-related forums revealed a weakness in social participation, even though health councils and Caps assemblies proved to be strong mechanisms in all three cases. User participation in discussions strengthens leadership and effective governance—an objective established by the World Health Organization (WHO) in its Mental Health Action Plan (16)—and aligns with a key principle of the Brazilian anti-asylum fight (“Nothing about us, without us?”) (27).

Another convergent critical aspect was the low regularity in health data collection, analysis, monitoring, and dissemination. Health surveillance is crucial for organizing and planning actions and for supporting decision-making. Although more common in primary health care, teams operating in specialized psychosocial care must also recognize mental health needs and territorial resources, and institutionalize the evaluation of their practices as an educational process. This will support the formulation of new care strategies aligned with user needs and articulated with the network (28). Strengthening information systems, data collection and analysis, and mental health research is also widely recommended by the World Health Organization (WHO) (15). 

The implementation and maintenance of health promotion and prevention strategies were hindered by the COVID-19 pandemic across the three cases. The impacts of the pandemic generated care disruptions in health systems worldwide (29), including a reduction in group activities (24). Nonetheless, evidence exists regarding the usability, safety, acceptability, satisfaction, and effectiveness of remote interventions—applied to varying degrees in all three cases—although evidence concerning process quality, cost-effectiveness, and long-term effects remains limited (30).

The intentional selection of municipalities with networks recognized for strong primary health care, low hospitalization rates, or participation in a Raps activation project enabled the identification of successful approaches—especially those centered on singular and territorial care—that are potentially transferable to other contexts. The findings suggest that improving integrality in the Raps requires increased investments in the production, integration, and dissemination of data, as well as in planning and evaluation, thereby strengthening the articulation of its components.

## Data Availability

The database used in this research is not publicly available in order to protect the privacy of study participants. However, an anonymized database may be made available by the corresponding author upon request.
