# Supplementary material for: Assessment of the integrality of the psychosocial care network: multiple case study, Southern Region of Brazil, 2021
Source: Epidemiol Serv Saude. 2026 Jul 27;35:e20240890. doi: 10.1590/S2237-96222026v35e20240890.en (PMC13686392; doi:10.1590/S2237-96222026v35e20240890.en)
Supplement: Translation

# Translation: Avaliação da integralidade da rede de atenção psicossocial: estudo de casos múltiplos, região Sul do Brasil, 2021

**DOI:** 10.1590/S2237-96222026v35e20240890.pt

**Authors:** Larissa Weber, Claudia Flemming Colussi

**Affiliations:** 1 Prefeitura Municipal de Florianópolis Florianópolis Florianópolis SC Brasil Prefeitura Municipal de Florianópolis, Florianópolis, SC, Brasil; 2 Universidade Federal de Santa Catarina Florianópolis Florianópolis SC Brasil Universidade Federal de Santa Catarina, Florianópolis, SC, Brasil

**Keywords:** Integralidade em Saúde, Avaliação em Saúde, Saúde Mental, Redes Comunitárias, Relato de caso

## Abstract

**Objetivo:**

Avaliar a integralidade das ações em saúde dos serviços da rede de atenção psicossocial (Raps) em três municípios da região Sul do Brasil com diferentes portes populacionais e variadas configurações da Raps.

**Método:**

Pesquisa avaliativa baseada em um estudo de casos múltiplos. Utilizou-se uma matriz de análise e julgamento com duas dimensões, seis subdimensões e 16 indicadores. Os dados primários provenientes de cinco entrevistas por município com gestor, profissionais e usuários, totalizando 15 entrevistas, foram triangulados com dados oriundos de observação direta e dados secundários, todos coletados em campo em 2021. Procedeu-se à análise de conteúdo dos dados qualitativos, enquanto os dados de natureza quantitativa foram submetidos à análise descritiva.

**Resultados:**

Os três casos apresentaram resultado satisfatório na dimensão “Elementos essenciais”. Esse achado expressou a viabilidade de implantação de ações qualificadas de cuidado territorial, como a oferta de acolhimento, a ênfase na singularidade e na autonomia dos usuários e a atenção direcionada aos familiares, qualquer que seja o tamanho da Raps dos municípios. Apenas o caso 3 foi bem avaliado na dimensão “Integrações”, na qual os outros dois obtiveram desempenho regular. A ocorrência de processos de trabalho afeitos à lógica do cuidado em rede em um cenário em que coexistem todos os componentes da Raps favoreceu o desempenho do caso 3 nessa dimensão.

**Conclusão:**

Sobressaiu-se positivamente a oferta de cuidado singular e territorial nos três casos. Mostrou-se necessário ainda aperfeiçoar a articulação dos componentes da Raps, sobretudo através de investimentos na produção, integração e difusão de dados.

**Aspectos éticos**

A presente pesquisa respeitou os princípios éticos, obtendo os seguintes dados de aprovação:

Comitês de ética em pesquisa Números dos pareceres, Datas de aprovação, Certificado de apresentação de apreciação ética

Universidade Federal de Santa Catarina 4.624.644, 31/3/2021, 43341921.5.0000.0121

Secretaria Municipal de Saúde de Curitiba 4.791.120, 18/6/2021, 43341921.5.3001.0101

Registro do consentimento livre e esclarecido Obtido de todos os participantes antes da coleta.

## Introdução

A integralidade pressupõe o usuário como elemento central do cuidado em saúde e requer a adoção de práticas colaborativas e coordenadas para promover, prevenir, restaurar a saúde e reabilitar os sujeitos (1), reconhecendo a interdependência entre saúde mental, saúde física e bem-estar social (2). No campo da atenção psicossocial, o cuidado integral envolve a elaboração de projetos terapêuticos singulares que compreendam os indivíduos nos contextos familiar, social, político e histórico e a composição de uma rede de cuidados integrada e qualificada (3). 

A rede de atenção psicossocial (Raps), que consiste em uma rede de atenção à saúde temática (4), foi instituída no Brasil com base nesse preceito. Ela busca ampliar o acesso dos usuários, diversificar as estratégias de cuidado e articular os pontos de atenção. Estes estão agrupados nos seguintes componentes: atenção básica, atenção psicossocial especializada, atenção de urgência e emergência, atenção hospitalar, atenção residencial de caráter transitório, desinstitucionalização e reabilitação psicossocial (5).

Esse cenário de reorientação do modelo de cuidado carece ainda de pesquisas avaliativas, especialmente voltadas à integração entre os serviços da Raps (6,7). A proposição de avaliações no âmbito das redes de saúde é ainda incipiente e desafiadora, pela complexidade imposta pela análise das interações entre múltiplos atores e serviços (8). 

Este estudo considerou o potencial da avaliação para embasar a tomada de decisão e aprimorar ações, serviços, redes e sistemas de saúde. Considerou também que a organização da Raps não se dará de forma padronizada no território brasileiro, atentando para as particularidades municipais e regionais. O objetivo deste estudo foi avaliar a integralidade das ações em saúde dos serviços da Raps em três municípios da região Sul do Brasil com diferentes portes populacionais e configurações da Raps.

## Métodos

### Delineamento

Trata-se de estudo transversal, caracterizado por pesquisa avaliativa baseada em estudo de casos múltiplos (9) com três municípios. A avaliação deu-se através da aplicação de uma matriz de análise e julgamento desenvolvida para avaliar as ações e os serviços da atenção psicossocial sob a perspectiva da integralidade nos municípios, a fim de ofertar meios para qualificar o cuidado no campo, a gestão e a participação social. Este estudo não se propôs a avaliar o instrumento em si (a matriz), mas sim os três casos selecionados. 

A matriz foi composta por duas dimensões (D) e seis subdimensões (S): “D1 Elementos essenciais”, subdividida em “S1 Estrutura”, “S2 Cuidado” e “S3 Gestão”; e “D2 Integrações”, subdividida em “S4 Integração dos cuidados”, “S5 Integração funcional” e “S6 Integração normativa”, totalizando 16 indicadores e 52 medidas (10). A proposta original sofreu algumas alterações: fusão das medidas “M4 Serviços de atenção básica” acessíveis a pessoas com deficiência e “M5 Centro de atenção psicossocial (Caps)” acessíveis a pessoas com deficiência, pois os municípios seriam penalizados na avaliação se quaisquer dos serviços não se mostrassem acessíveis; fusão da “M18 Elaboração do Plano Terapêutico Singular na atenção básica dos casos acompanhados em matriciamento” e “M19 Programação de visita domiciliar pelas equipes de atenção básica dos casos discutidos em matriciamento”, motivada pelo entendimento de que a programação das visitas compõe os planos terapêuticos singulares, não havendo necessidade de separar essa ação em duas medidas; e exclusão da “M17 Proporção de usuários com tratamento centrado na medicação”, visto que não seria viável extrair esse dado a partir dos registros de atendimento.

### Contexto

A seleção dos casos levou em conta o porte populacional e a composição da Raps dos municípios (Tabela 1). Considerou-se ainda o preenchimento de, ao menos, dois dos critérios a seguir.

**Tabela 1:** Pré-requisitos para seleção dos casos da rede de atenção psicossocial (Raps). Região Sul do Brasil, 2021 (n=3)

Pré requisitos	Caso 1	Caso 2	Caso 3
Tamanho da população (habitantes)	Até 20 mil	70 mil-150 mil	>150 mil
Composição da Raps	Ofertar cuidado em saúde mental oferecida majoritariamente pela atenção básica. Não possuir centro de atenção psicossocial (Caps).	Possuir ao menos um Caps e outro serviço entre os seguintes componentes da Raps: atenção residencial de caráter transitório, reabilitação ou desinstitucionalização.	Possuir todos os componentes da Raps.

Obter resultado satisfatório na pesquisa “Avaliação das ações em saúde mental na atenção básica”, que utilizou o banco de dados do Programa Nacional de Melhoria do Acesso e da Qualidade da Atenção Básica (11). Os municípios foram divididos de acordo com os portes populacionais para, em seguida, proceder-se ao cálculo do valor correspondente ao quartil superior em cada um dos três grupos, com o propósito de identificar os mais bem avaliados. Critério obrigatório para seleção do caso 1.
Participar do projeto “Percursos formativos na Raps: intercâmbio entre experiências”, promovido pelo Ministério da Saúde, cujo objetivo era fomentar o desenvolvimento da Raps mediante a realização de intercâmbio de profissionais entre diferentes municípios e a contratação de ativadores de redes.
Apresentar baixas taxas de internações decorrentes de agravos à saúde mental entre setembro de 2019 e agosto de 2020. Para calcular a taxa, extraiu-se do Departamento de Informática do Sistema Único de Saúde a população estimada dos municípios da região Sul em 2019 e o total de internações decorrentes de transtornos mentais e comportamentais devido ao uso de álcool ou outras substâncias psicoativas; esquizofrenia, transtornos esquizotípicos e delirantes; transtornos de humor; transtornos neuróticos e relacionados com estresse; deficiência intelectual; e outros transtornos mentais e comportamentais. Os municípios foram separados por portes, visando identificar aqueles que integravam o quartil inferior em cada grupo, o que os tornava elegíveis.

Aplicados tais pré-requisitos e critérios, chegaram-se a 24 municípios da região Sul elegíveis para o caso 1; quatro para o caso 2; e quatro para o caso 3. A seleção final levou em conta a representação dos três estados, razão pela qual se optou por escolher um caso por estado.

### Participantes

Participaram um gestor, dois profissionais e dois usuários da Raps em cada um dos três municípios, o que totalizou 15 participantes. Gestores e profissionais foram indicados pela gestão das secretarias municipais de saúde, com exceção de uma profissional, selecionada por conveniência. Os usuários foram indicados pelos gestores e profissionais entrevistados.

### Variáveis

Foram utilizadas as variáveis de desfecho e as explicativas. As de desfecho incluíram a integralidade das ações e dos serviços da Raps nos municípios estudados, conforme pontuação atribuída pela matriz. As variáveis explicativas consideraram o porte populacional do município (pequeno, médio, grande) e a composição da Raps local (presença ou não de Caps e outros pontos de atenção).

### Fontes de dados e mensuração

A coleta dos dados primários incluiu entrevistas e observações, ocorridas em julho e agosto de 2021. No caso 3, foi designada uma região da cidade para a coleta dos dados. Todos os locais visitados eram serviços da administração direta, habilitados e com equipes completas no momento da coleta.

As entrevistas semiestruturadas foram realizadas majoritariamente de forma presencial (n=14). A condução deu-se por uma única pesquisadora, a partir de um roteiro baseado nos elementos da matriz. Todas foram gravadas e transcritas, com a concordância dos participantes, identificados pela categoria (G=gestor, P=profissional, U=usuário), seguida por um número de 1 a 6.

A observação, do tipo sistemática, foi realizada pela mesma pesquisadora que conduziu as entrevistas e ocorreu nos mesmos locais, totalizando três turnos por município. Observaram-se, entre outros aspectos, a estrutura, as interações e as informações divulgadas na recepção dos serviços. Os dados foram registrados em diário de campo e utilizados como complemento na análise de algumas medidas.

Os dados secundários foram coletados em campo por meio de documentos do biênio 2020-2021 relativos aos componentes da Raps nos municípios, como protocolos, fluxogramas, normativas, atas de reuniões, memorandos e instrumentos de planejamento. Os documentos foram representados pela letra D, seguida por um número de 1 a 5.

### Viés

A indicação dos participantes pode ter privilegiado profissionais e usuários mais engajados ou com experiências positivas, com possível viés de seleção. No caso dos usuários, considera-se que o viés de gratidão pode tê-los inibido a tecer críticas aos serviços. 

O uso de entrevistas gravadas e observações presenciais pode gerar respostas socialmente desejáveis que ocultem as fragilidades dos serviços, principalmente em profissionais e gestores, o que causa viés de informação. 

A realização das entrevistas e observações por uma única pesquisadora pode gerar viés de interpretação ou condução, mesmo com roteiros padronizados. Buscou-se minimizar esse potencial viés com triangulação e validação dos dados.

### Tamanho do estudo

O estudo envolveu três municípios da região Sul do Brasil e teve 15 participantes, cinco por município.

### Métodos estatísticos

Os dados de natureza qualitativa foram submetidos à análise de conteúdo (12), considerando as subdimensões e dimensões da matriz como categorias previamente definidas. Os dados quantitativos foram transferidos para planilhas em Excel, onde se procedeu à análise descritiva. Considerando a diversidade de fontes e formas de coleta de dados, optou-se pela triangulação metodológica como modelo de análise (13), que resultou na análise de cada um dos casos separadamente e na análise imbricada, na qual foram apreciados aspectos semelhantes e divergentes entre eles.

Na matriz, a soma da pontuação das medidas resultou na pontuação de cada indicador. A pontuação obtida (PO) foi dividida pela pontuação máxima possível do indicador (PM), mediante a aplicação da fórmula “escore=(∑PO/∑PM)X100”. Ao valor percentual obtido, conferiu-se o seguinte juízo de valor: até 49% foi ruim; de 50%-74% foi regular; e igual ou acima de 75% foi bom. Para a análise das subdimensões, atribuíram-se aos indicadores os seguintes escores: 1 (um) para bom; 0,5 (meio) para regular; e 0 (zero) para ruim. O valor dos indicadores das subdimensões foi somado para obter-se a pontuação delas. Aplicou-se a mesma fórmula e o mesmo juízo de valor utilizados para os indicadores. Tal procedimento também foi feito em relação às dimensões – a partir da soma da pontuação de cada subdimensão e da aplicação da fórmula, atribuiu-se o juízo de valor final.

## Resultados

A Tabela 2 caracteriza profissionais e gestores participantes. Quanto aos usuários, tratava-se de quatro homens e duas mulheres, em tratamento na rede há pelo menos um ano e meio. Foram caracterizados os casos quanto a dados demográficos (14) e composição da Raps (Tabela 3).

**Tabela 2:** Caracterização dos profissionais de saúde e dos gestores entrevistados nos três municípios. Região Sul do Brasil, 2021

Codificação	Categoria	Sexo	Formação	Local de atuação	Tempo de serviço no município
G1	Gestor	Feminino	Terapeuta ocupacional	Coordenadora do centro de atenção psicossocial (Caps)	11 anos
G2	Gestor	Feminino	Psicóloga	Coordenadora de saúde mental	19 anos
G3	Gestor	Feminino	Enfermeira	Secretária municipal de saúde	20 anos
P1	Profissional de saúde	Feminino	Terapeuta ocupacional	Caps II e Caps ad	1 ano e 7 meses
P2	Profissional de saúde	Feminino	Psicóloga	Núcleo de apoio à saúde da família (Nasf)	19 anos
P3	Profissional de saúde	Feminino	Técnica de enfermagem	Caps II	8 anos
P4	Profissional de saúde	Feminino	Enfermeira	Unidade básica de saúde	3 anos
P5	Profissional de saúde	Masculino	Psicólogo	Nasf	2 anos
P6	Profissional de saúde	Feminino	Psicóloga e acupunturista	Estratégia de saúde da família	7 anos

**Tabela 3:** Características demográficas e composição da rede de atenção psicossocial (Raps) dos três casos avaliados. Região Sul do Brasil, 2023

Características		Caso 1	Caso 2	Caso 3
População (habitantes)		11.648	92.924	1.773.733
Produto interno bruto per capita		R$ 45.245,79	R$ 59.309,03	R$ 45.318,46
Índice de desenvolvimento humano municipal		0,737	0,730	0,823
Composição da Raps	4 estratégias de saúde da família (ESF) 1 unidade básica de saúde (UBS) 1 núcleo ampliado de saúde da família (Nasf) 1 centro de especialidades 1 centro de atenção psicossocial (Caps) regional (localizado em outro município, não avaliado neste estudo)		6 ESF 7 UBS 1 Caps II 1 Caps II ad 1 Caps II IJ 1 policlínica 1 serviço de pronto atendimento 1 serviço de atendimento móvel de urgência 1 hospital geral 1 serviço residencial terapêutico (conveniado, localizado em outro município)	55 UBS 53 UBS (com saúde da família) 39 Nasf 4 consultórios na rua 4 Caps II adulto 6 Caps III adulto 2 Caps II IJ 1 Caps III IJ 1 ambulatório de saúde mental 1 ambulatório para transtornos do espectro autista 9 unidades de pronto atendimento 1 unidade de estabilização psiquiátrica 1 hospital psiquiátrico 5 serviços residenciais terapêuticos

A partir da análise dos dados oriundos das entrevistas, das observações e dos documentos, os 3 casos alcançaram desempenho satisfatório na dimensão “D1 Elementos essenciais”. A dimensão “D2 Integrações” foi considerada satisfatória no caso 3 e regular nos demais. 

Apresentou-se a pontuação obtida por cada município após aplicação da matriz (Tabela 4). O caso 1 obteve desempenho máximo na “S1 Estrutura”, “S2 Cuidado” e “S6 Integração normativa” e teve expressiva pontuação nas medidas referentes à atenção básica (14 de 15).

**Tabela 4:** Resultado da avaliação dos três casos a partir da pontuação dos indicadores (I), das subdimensões (S) e das dimensões (D) da matriz de análise e julgamento. Região Sul do Brasil, 2021

Indicadores	Pontuação dos casos			Subdimensões	Pontuação dos casos			Dimensões	Pontuação dos casos		
	1	2	3		1	2	3		1	2	3
I1 Recursos físicos	100	58	75	S1 Estrutura	100	75	100	D1 Elementos essenciais	100	100	100
I2 Recursos humanos	100	100	100								
I3 Acolhimento	100	100	100	S2 Cuidado	100	100	100				
I4 Acompanhamento diversificado	100	100	100								
I5 Atenção à crise	100	100	100								
I6 Educação Permanente em Saúde	67	60	80	S3 Gestão	83	83	100				
I7 Instrumentos de gestão	100	100	100								
I8 Controle social	100	75	100								
I9 Corresponsabili-dade pelos casos	80	100	67	S4 Integração dos cuidados	83	100	83	D2 Integrações	67	67	100
I10 Promoção e pre-venção no território	83	83	83								
I11 Reabilitação no território	67	83	100								
I12 Vigilância em saúde	67	62	62	S5 Integração funcional	33	50	83				
I13 Gestão da clínica	50	75	75								
I14 Articulação da rede	37	37	75								
I15 Regulação	100	33	100	S6 Integração normativa	100	50	100				
I16 Informatização	75	50	75								

Os investimentos em agentes comunitários de saúde – “a gente consegue ter essa integralidade através do que o agente de saúde nos traz de informação” (G3) –, na implantação das equipes multidisciplinares – “Nasf faz uma diferença maravilhosa, (...) com essa proposta de fazer as ligações, (...) desenho de fluxo, encaminhamento, matriciamento” (G3) – e nas práticas integrativas e complementares – “ofertar (...) uma auriculoterapia, um reiki” (G3) – explicitaram o bom desempenho nesse conjunto de medidas.

Por outro lado, reconheceu-se dificuldade na formalização de fóruns e reuniões no município (intra e intersetoriais), haja vista o baixo desempenho no “I14 Articulação da rede”, que refletiu negativamente na S5 Integração funcional. Tampouco as reuniões de equipe na atenção básica estavam ocorrendo durante a coleta de dados.

Houve percepção de que se poderia agir mais preventivamente nas discussões de caso – “Geralmente é feito quando as coisas se complicam (...) a gente às vezes chega tarde” (G3). A designação de espaços formais para trocas, por vezes, foi minimizada pela proximidade entre os profissionais – “poderia ter alguma reunião, (...) uma integração maior. Claro que pelo contato pessoal que a gente tem às vezes facilita” (P5). O eventual desalinhamento entre os profissionais pôde comprometer o cuidado – “às vezes deixa a desejar um pouco (...), porque um diz uma coisa, (...) a outra já não acha que é aquilo” (U5).

Foi levantada a necessidade de maiores investimentos na área infanto-juvenil – “não tem escolinha de futebol, não tem qualquer outra coisa que possa envolver essas crianças. (...) não tem como ter pessoas com uma mente saudável, se não se trata saudável desde a infância” (U6).

O caso 2 destacou-se pelo desempenho no “I9 Corresponsabilidade pelos casos”, na “S2 Cuidado” e na “S4 Integração dos cuidados”. Apesar da ausência de Nasf (terminologia mantida neste estudo, apesar da descontinuidade do serviço, considerando o período da coleta de dados), os serviços de atenção básica do município foram matriciados pelos Caps, e algumas equipes contavam com psicólogos(as) e estagiários(as) de psicologia. O matriciamento foi valorizado pelas equipes tanto na dimensão assistencial – “eles pedem para falar com o técnico que vai sempre junto no matriciamento, porque sabem que tá apropriado” (P3) – como pelo papel técnico-pedagógico – “funcionava super bem, porque se discutiam casos, tiravam dúvidas, às vezes inseria uma medicação na discussão do caso ou fazia uma interconsulta” (P4).

A rede teve seu desempenho prejudicado pela baixa cobertura de atenção básica (43,0%). Se contabilizadas apenas as ESF, a cobertura é ainda menor (21,0%). A provável sobrecarga decorrente desse fato refletiu em processos de trabalho que desprestigiaram ações de promoção e prevenção em saúde. O município previu aumentar a cobertura da atenção básica para 60,0% e das ESF para 40,0% até 2025, além de implementar grupos de educação em saúde nas unidades (D2). A abordagem à saúde mental na atenção básica careceu também de mais incentivos – “a gente conseguiu avançar muito na constituição dos Caps como política pública no município, mas a atenção primária tem que avançar muito ainda em discussão de saúde mental” (G2).

As equipes dos Caps apresentaram histórico de iniciativas voltadas à articulação em rede – “nunca foi muito fácil a construção da Raps, essa articulação de rede parece muitas vezes que é um movimento de uma direção única (...) centrado nos Caps” (G2). Entretanto, a ausência de um calendário institucional de reuniões de rede no momento da coleta de dados, mesmo que justificada pela pandemia, sinalizou uma frágil designação dos fluxos de trabalho e dos papéis dos atores. Isso ficou evidente pela baixa pontuação de “I15 Regulação” e “I14 Articulação da rede” – “Não há nada formal, escrito, que se faça isso, faça aquilo” (P3). Destacou-se a importância de não engessar os fluxos para que não se perdesse de vista a singularidade dos casos – “O caso não existe no papel, o caso existe discutido em equipe” (P3).

O caso 3 foi avaliado como satisfatório em todas as subdimensões e dimensões, com nota máxima nas 3 subdimensões da “D1 Elementos essenciais” e da “S6 Integração normativa”. O bom desempenho foi impulsionado pelo trabalho de base territorial e longitudinal: “a territorialidade, ela fundamenta muito do trabalho, (...) amplifica a nossa capacidade de atendimento, (...) o vínculo das equipes com o território” (P2). Profissionais da psicologia e psiquiatria foram designados como apoiadores da atenção básica no Plano Municipal de Saúde, desenvolvendo ações intra e intersetoriais em território definido (D3). Sobressaiu-se também a ênfase na singularidade e autonomia dos usuários – “Já tiramos alguma medicação, diminuímos outra, (...) na próxima consulta vou falar que não quero mais remédio para dormir. (...) vou ficar com o estabilizador de humor, que ainda acho necessário” (U1).

O município dispôs de um documento norteador do cuidado em saúde mental, o qual contemplou as atribuições de cada serviço da Raps, os critérios de encaminhamento e as diretrizes clínicas (D4). Houve também diretrizes institucionais específicas para os Caps, alinhadas ao modelo da atenção psicossocial (D5). 

A coexistência de mecanismos formais e informais de articulação intra e intersetorial impactou positivamente a integralidade: “uma coisa bem prática de ver essa integralidade do cuidado é justamente esse apoio da rede” (P1). Os desempenhos menos satisfatórios do caso, relacionados aos indicadores da “S4 Integração dos cuidados” e “S5 Integração funcional”, sugeriram que o município fortalecesse a corresponsabilidade e vigilância em saúde.

A análise imbricada privilegiou a comparação entre os casos e salientou aspectos convergentes e divergentes. O bom desempenho dos casos na maioria das subdimensões justificou o predomínio de aspectos favoráveis sobre desfavoráveis (Tabela 5).

**Tabela 5:** Aspectos convergentes e divergentes entre os três casos avaliados. Região Sul do Brasil, 2021

Aspectos convergentes	
Favoráveis	Desfavoráveis
Existência de equipe de apoio matricial em saúde mental na atenção básica. Composição das equipes mínimas dos centros de atenção psicossocial (Caps) alinhadas às portarias do Ministério da Saúde (não se aplica ao caso 1). Acolhimento à demanda espontânea nos Caps e serviços de atenção básica. Elaboração de Plano Terapêutico Singular para todos os usuários nos Caps e casos acompanhados em matriciamento na atenção básica. Implantação de atendimento remoto. Oferta de grupos e oficinas nos Caps e na atenção básica. Processos de trabalho que contemplem a atenção à crise e a busca ativa de casos faltosos graves nos Caps (não se aplica ao caso 1). Orientação a familiares. Oferta de atendimento interdisciplinar na internação hospitalar. Metas para a saúde mental no Plano Municipal de Saúde. Realização de assembleias nos Caps (não se aplica ao caso 1). Pactuações sobre saúde mental no Conselho Municipal de Saúde. Consultas e visitas domiciliares em conjunto com profissionais de saúde mental e da atenção básica unidade. Realização de campanhas de prevenção e atividades de Educação em Saúde. Existência de centros de convivência ou espaços afins. Definição de critérios para encaminhamento ao Caps.	Mapeamento dos fatores de risco e proteção nos territórios não é feito com regularidade. Dificuldade de incluir usuários nas reuniões de rede. Prontuário eletrônico não integra todos os pontos de atenção da rede de atenção psicossocial.
Aspectos divergentes	
Favoráveis	Desfavoráveis
Casos 1 e 2: equipes de referência acompanham os casos internados. Casos 1 e 3: estabelecimento de critérios para acionar o serviço de atendimento móvel de urgência e para indicar internação hospitalar. Casos 2 e 3: existência de atividades de trabalho e geração de renda na rede e de projetos para inserção/acompanhamento de usuários no mercado de trabalho. Acompanhamento dos egressos de longa internação.	Casos 1 e 2: dificuldade de sistematizar horários de educação permanente em saúde e de reuniões de rede. Casos 2 e 3: acessibilidade comprometida nos Caps e em algumas unidades de atenção básica.

## Discussão

A utilização da matriz como instrumento avaliativo retratou forças e fragilidades da integralidade no contexto da Raps de três municípios da região Sul do Brasil, enquanto a triangulação demonstrou convergência no conjunto dos dados analisados. Foi possível identificar que os casos 1 e 2 obtiveram resultado satisfatório na “D1 Elementos essenciais” e regular na “D2 Integrações”, enquanto o caso 3 obteve bom desempenho nas duas dimensões.

Foram limitações deste estudo a impossibilidade de visitar todos os serviços dos municípios; a representação de profissionais e gestores restrita a três núcleos profissionais; e a coleta de dados durante a pandemia de covid-19, a qual se atribui também o cancelamento do estudo-piloto.

O bom desempenho obtido pelos três casos na D1 corrobora a viabilidade de ofertar cuidado qualificado nos territórios, qualquer que seja o tamanho da Raps dos municípios. O acolhimento, a ênfase na singularidade dos usuários e a atenção aos familiares consistem em práticas recomendadas globalmente (15-16) e que foram identificadas nos diferentes cenários avaliados. Sua disseminação foi influenciada pela Política Nacional de Saúde Mental, cujas normativas instruem gestores e trabalhadores nessa direção (5,17-18).

Restou evidente a potência da coordenação do cuidado e ordenação da rede pelas equipes de atenção básica nos casos 1 e 3, em consonância com a Política Nacional de Atenção Básica (19). No caso 2, reconheceu-se o deslocamento de algumas atribuições da atenção básica para os Caps. Isso reflete a realidade de outros municípios (7,20-21) e encontra amparo na Portaria nº 336/2002, que conferiu aos Caps a responsabilidade pela organização da demanda e da rede de cuidados em saúde mental no território (18). Posteriormente, a Portaria da Raps estabeleceu que a ordenação do cuidado pode ser atribuída aos Caps ou à atenção básica (5). 

Outra convergência favorável à integralidade identificada nos casos 1 e 3 foi o estabelecimento de critérios bem definidos para encaminhamentos na rede, além da implantação de uma regulação em saúde mental. Os protocolos e fluxos qualificam acolhimentos e encaminhamentos, orientam a conduta, aprimoram a articulação entre os serviços e evitam internações desnecessárias (22), constituindo estratégia fundamental para a integração da rede (23). Haja vista as diferenças de estrutura entre os dois casos, trata-se de um processo de trabalho que não depende de Raps robusta para ser executado. A adoção desses instrumentos cresceu após a pandemia, inclusive para orientações quanto ao acesso aos cuidado em saúde mental (24).

A diversificação do cuidado foi mais expressiva nos casos 2 e 3, impulsionada pelas ações voltadas à reabilitação psicossocial e à desinstitucionalização, através de espaços de convivência e geração de renda. Tais serviços instigam a autonomia, cidadania e inclusão social – intervenções que tem tanta importância quanto o cuidado assistencial para sujeitos em sofrimento psíquico (7).

Se, por um lado, municípios com redes mais enxutas podem dispor de menos profissionais e serviços para efetivar ações desse caráter, por outro, a inexistência de recursos especializados em saúde mental reforça a participação ativa dos gestores nas pactuações da sua região de saúde, a exemplo do que foi constatado no caso 1. A regionalização aumenta o acesso e a eficiência do sistema de saúde, integrando a oferta de serviços através da ação cooperativa e continuada entre municípios (25). Por meio dela, firmou-se um convênio entre o caso 1 e município vizinho, que viabilizou o Caps regional.

A ocorrência de processos de trabalho afeitos à lógica do cuidado em rede em um cenário em que coexistem todos os componentes da Raps favoreceu o desempenho do caso 3, o mais bem sucedido nesta avaliação. Entre esses processos, a sistematização de reuniões e fóruns de articulação favoreceu o entrosamento entre profissionais, resultando na melhor avaliação da “D2 Integrações”. O trabalho colaborativo é especialmente profícuo no contexto da Raps por romper com o modelo assistencial fragmentado, valorizando as competências de cada profissional, a comunicação horizontal e o compartilhamento da tomada de decisões (26).

A ausência de usuários nos referidos fóruns relacionados à atenção psicossocial descortinou uma fragilidade na participação social, ainda que os conselhos de saúde e as assembleias dos Caps tenham se mostrado potentes nos três casos. A presença dos usuários nas discussões fortalece a liderança e a governança eficaz, objetivo estabelecido pela Organização Mundial da Saúde em seu pelo Plano de Ação sobre Saúde Mental (16), além de atender a um dos lemas da luta antimanicomial (“Nada sobre nós sem nós”) (27).

Outro aspecto crítico convergente consistiu na baixa regularidade na coleta, na análise, no monitoramento e na divulgação de informações de saúde. A vigilância em saúde é crucial para a organização e o planejamento das ações, embasando a tomada de decisões. Embora seja mais comum no domínio da atenção básica, equipes atuantes na atenção psicossocial especializada também devem reconhecer as demandas em saúde mental e os recursos dos territórios, assim como institucionalizar a avaliação das suas práticas como um processo educativo. Este subsidiará a formulação de novas estratégias de cuidado, alinhadas às necessidades dos usuários, em articulação com a rede (28). O fortalecimento dos sistemas de informação, da coleta e análise de dados e das pesquisas em saúde mental também é largamente recomendado pela Organização Mundial da Saúde (15). 

A implementação e manutenção de estratégias de promoção e prevenção da saúde viu-se prejudicada pela pandemia de covid-19 nos três casos. Os impactos impostos pela pandemia geraram desassistência em diferentes sistemas de saúde ao redor do mundo (29), incluindo redução na oferta de atividades grupais (24). Ressalta-se, contudo, a existência de evidências sobre a usabilidade, segurança, aceitação, satisfação e eficácia das intervenções remotas – aplicadas em maior ou menor grau nos três casos – conquanto as evidências sobre a qualidade do processo, a relação custo-eficácia e os efeitos em longo prazo sejam limitados (30).

A seleção intencional de municípios cujas redes se destacavam pela qualidade da atenção básica, pela baixa proporção de internações ou pela participação em um projeto de ativação da Raps identificou aspectos exitosos, especialmente voltados ao cuidado singular e territorial, replicáveis em outras realidades. Os achados sugerem que o aperfeiçoamento da integralidade na Raps perpassa a injeção de mais investimentos na produção, integração e difusão de dados, assim como no planejamento e na avaliação, impulsionando a articulação dos seus componentes.

## Data Availability

O banco de dados utilizados nesta pesquisa não está disponível publicamente, a fim de proteger a privacidade dos participantes do estudo. Entretanto, um banco anonimizado pode ser disponibilizado pelo autor correspondente mediante solicitação.
